# Combined Effects of Radiative and Evaporative Cooling on Fruit Preservation under Solar Radiation: Sunburn Resistance and Temperature Stabilization

**DOI:** 10.1021/acsami.2c11349

**Published:** 2022-09-29

**Authors:** Liang Xu, Da-Wen Sun, You Tian, Libin Sun, Tianhao Fan, Zhiwei Zhu

**Affiliations:** † School of Food Science and Engineering, South China University of Technology, Guangzhou 510641, China; ‡ Academy of Contemporary Food Engineering, South China University of Technology, Guangzhou Higher Education Mega Center, Guangzhou 510006, China; § Engineering and Technological Research Centre of Guangdong Province on Intelligent Sensing and Process Control of Cold Chain Foods, & Guangdong Province Engineering Laboratory for Intelligent Cold Chain Logistics Equipment for Agricultural Products, Guangzhou Higher Education Mega Centre, Guangzhou 510006, China; ∥ Food Refrigeration and Computerized Food Technology (FRCFT), Agriculture and Food Science Centre, University College Dublin, National University of Ireland, Belfield, Dublin D04 V1W8, Ireland

**Keywords:** passive cooling, hydrogel, nanocomposite, fruits, quality attributes, multivariate statistical analysis

## Abstract

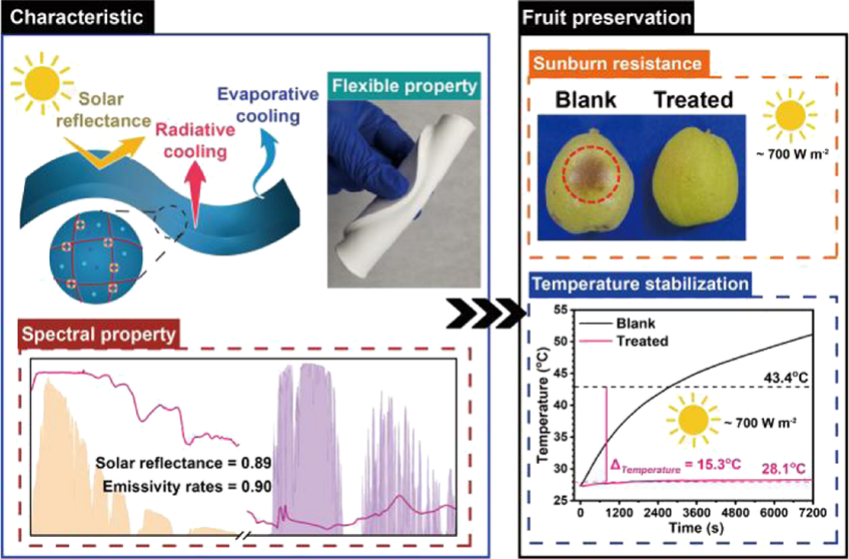

Excessive solar radiation and high temperature often cause considerable loss and waste of fruits during transportation, retail, and storage. In the current study, a natural deep eutectic solvent-based polyacrylamide/poly(vinyl alcohol) hydrogel with nanoparticles (NPs/NADES@PAAm/PVA) is developed for fruit quality protection from solar radiation and high-temperature stress by achieving the combined effect of radiative and evaporative cooling. NPs/NADES@PAAm/PVA presents an average solar reflectance of ∼0.89 and an average emittance at the atmospheric window of ∼0.90. Besides, NPs/NADES@PAAm/PVA possesses excellent flexibility, robust mechanical strength, and good swelling behavior. The fruit preservation experiments under sunlight demonstrate that the pear (*Pyrus sinkiangensis*) treated with NPs/NADES@PAAm/PVA can achieve an average temperature decrease of ∼15.3 °C after sun exposure compared with the blank, and its quality-related attributes, including color, total soluble solid, relative conductivity, and respiration rate, are similar to the fresh one. Multivariate data analyses, including principal component analysis and cluster analysis, further verify that the pear treated with NPs/NADES@PAAm/PVA possesses similar quality to the fresh one after sun exposure. Thus, NPs/NADES@PAAm/PVA has promising prospects for fruit transportation, retail, and storage under solar radiation in a low-operation-cost and sustainable manner.

## 1 Introduction

Food loss and waste cause huge economic losses and serious environmental problems.^1^ Globally, around 14% of food is lost between harvest and retail, while an estimated 17% of food is wasted in households, food service, and retail.^2^ In low- and middle-income countries, significant amounts of fruits are lost and wasted during transportation, retail, and storage, often due to inadequate refrigerated facilities and electricity shortages.^3^ Therefore, it is urgent to search for a nonelectricity method to decrease fruit loss and waste in low- and middle-income countries.

In less developed countries and regions, postharvest fruits often suffer from strong solar radiation and high temperature during transportation, retail, and storage. Excessive solar radiation and extreme temperature can cause photooxidative damage and heat stress, stimulating sunburn development and thus leading to quality deterioration and lowering consumer acceptability of fruits.^4^ Generally, when fruit tissues absorb excessive solar radiation, reactive oxygen species are produced, inducing sunburn symptoms on fruits, which exhibit some white patches or dark brown regions due to the degradation of pigments, such as anthocyanin, chlorophyll, and carotenoid.^5^ Apart from solar radiation, fruit surface temperature (FST) is another critical factor influencing the degree of sunburn, and fruits are more susceptible to sunburn at high ambient temperatures. Torres et al. established the threshold FSTs of apples as 46 and 52 °C for browning and necrosis, respectively. McClymont et al. determined the threshold FSTs of red-blushed pears as 47 and 50 °C for browning and necrosis, respectively.^6,7^ However, fruits often exceed these threshold FSTs when they are exposed to sunlight. Besides, the high temperature promotes the metabolic activity of fruit, which is adverse to fruit preservation. Therefore, it is necessary to research effective sunburn resistance and temperature stabilization technologies.

Currently, some strategies to mitigate solar radiation damages are reported, including calcium carbonate spray,^8^ water spray,^9^ and sunshade.^10^ Although the spraying of fruits with protectants efficiently reduces sunburn, changes in organoleptic characteristics may affect consumer acceptability. Water spray can decrease the FST, but solar radiation can still affect the fruits. The sunshade is practical to reduce the sunlight effect, but the fruits still suffer from heat stress due to the high ambient temperature. Fortunately, with the development of materials science and nanotechnology, passive cooling technologies have emerged as an innovative approach to solar regulation and thermal management.^11−15^

Passive cooling technologies, including natural ventilation, microclimate, radiative cooling, and evaporative cooling,^11^ have aroused wide attention recently because of their cooling ability without any electricity input. Among these, radiative cooling transfers heat to a cold source (∼3 K) in outer space by emitting infrared radiation through the atmospheric window (8–13 μm). Combined with high solar reflectance enables the applications of radiative cooling materials under solar radiation, which is broadly studied in water cooling,^16^ ice preservation,^17^ photovoltaic panels,^18^ building cooling,^19^*etc*. Although radiative cooling can be achieved without energy consumption, its cooling performance cannot meet actual application requirements for fruit preservation. Moreover, evaporative cooling, which exploits water as the heat sink to take away a large amount of heat during evaporation, is widely explored in batteries,^20^ photovoltaic panels,^21^ cooling buildings,^22^ and garments.^23^ Under these circumstances, radiative and evaporative cooling could be employed as mutually supplemental cooling technology. However, studies on sunburn prevention and fruit preservation under solar radiation enabled by combining radiative and evaporative cooling technology have rarely been reported.

Therefore, the current study aims to develop a natural deep eutectic solvent-based polyacrylamide/poly(vinyl alcohol) hydrogel with nanoparticles (NPs/NADES@PAAm/PVA) for fruit preservation under solar radiation through radiative and evaporative cooling effects. In this study, a nanocomposite hydrogel with high solar reflectance and atmospheric emittance is developed by a one-pot synthesis method. After water absorption, the hydrogel can achieve subambient temperature under sunlight, contributed by the combined effects of radiative and evaporative cooling. To the best of our knowledge, this is the first study to report a nanocomposite hydrogel concerning the combined effects of radiative and evaporative cooling for fruit preservation under radiation through sun-proof and temperature stabilization functions. It is expected that this study should provide a low-operation-cost and sustainable cooling approach for fruit transportation, retail, and storage in less developed countries and regions.

## 2 Results and Discussion

### 2.1 Fabrication and Characterization of NPs/NADES@PAAm/PVA

**Figure 1:**
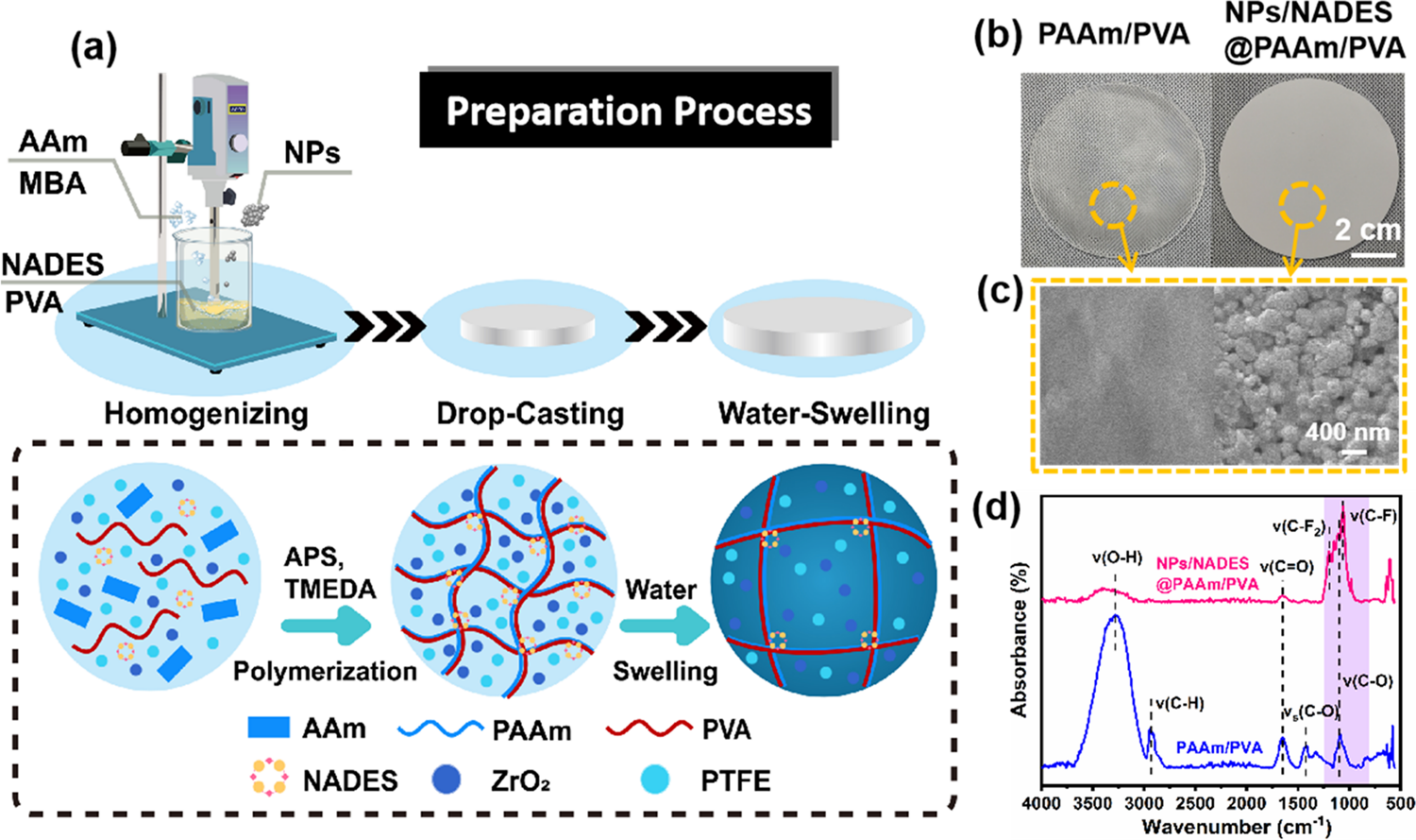
(a) Schematic diagram showing the preparation process of nanoparticles composite hydrogel. (b) Photographs showing PAAm/PVA and NPs/NADES@PAAm/PVA and their corresponding surface. (c) Scanning electron microscopy (SEM) images and (d) Fourier transform infrared (FTIR) spectra.

The fabrication strategy of NPs/NADES@PAAm/PVA is shown in Figure 1a. First, the natural deep eutectic solvent (NADES) is facilely prepared by stirring d-sorbitol (Sor) and l-proline (Pro) until a transparent liquid is obtained. Afterward, acrylamide (AAm), *N*,*N*′-methylenebisacrylamide (MBA), ammonium persulphate (APS), and NADES are mixed with a poly(vinyl alcohol) (PVA) solution to obtain a mother liquor, and high concentrations of nanoparticles (NPs) are dispersed in the mother liquor. As shown in Figure S1a, the partial NPs were agglomerated in the mother liquor by hand stirring, while the NPs were uniformly dispersed by homogenization, indicating that homogenization treatment is necessary. Noteworthily, since the NPs tend to accumulate at the bottom of the solution due to gravity, PVA as an emulsifier can stabilize the NPs in the hydrogel precursor after homogenization, which is an important step in achieving a well-dispersed precursor. After mixing *N*,*N*,*N*′,*N*′-tetramethylethylenediaminee (TMEDA) with the hydrogel precursor, AAm cross-links with MBA to form polyacrylamide (PAAm) and the PVA chains with abundant hydroxyl groups tightly bond with the amide groups in PAAm by forming hydrogen bonds to achieve a PAAm/PVA hydrogel with interpenetrating network structure. Noteworthily, the cross-linking reaction of the hydrogel precursor happens before casting due to its short reaction time, which is unfavorable for hydrogel fabrication. In contrast, the addition of NADES to the hydrogel precursor can delay the cross-linking reaction of the hydrogel. It is due to the fact that NADES as the donor and acceptor of hydrogen bonds can interact with AAm, PVA, and water molecules through hydrogen-bonding forces, which is the key for NPs/NADES@PAAm/PVA fabrication.^24^ After the cross-linking reaction, NPs/NADES@PAAm/PVA absorbs and stores water by immersion in water, which can exploit water as the heat sink to dissipate heat during water evaporation.

For achieving a desired daytime radiative cooling performance, high solar reflectance of NPs/NADES@PAAm/PVA is necessary due to the high solar irradiation intensity.^25^ As such, Zirconium dioxide nanoparticles (ZrO_2_ NPs) were selected to enhance the solar reflectance of NPs/NADES@PAAm/PVA due to their wide optical band gaps (5.4 eV, λ ∼ 0.227 μm).^26^ Apart from solar reflectance, high thermal emittance in the atmospheric window is also required, which can usually be enhanced by silicon dioxide (SiO_2_) particles due to their strong phonon polariton resonances in the atmospheric window.^27^ However, the hydrogel precursor with high concentrations of SiO_2_ particles is highly viscous, hindering the liquid-casting process. To overcome this challenge, poly(tetrafluoroethylene) nanoparticles (PTFE NPs), as lubricating agents, were selected to replace the SiO_2_ particles due to their chemical inertness and low surface energy, increasing the NP loading capacity of the hydrogel. Meanwhile, PTFE NPs are a difunctional radiative cooling material with weak absorptance in the solar spectrum and strong emission in the atmospheric window.^28^ Furthermore, the particle sizes of ZrO_2_ and PTFE NPs were optimized by Mie theory with Monte Carlo simulation,^26^ and ∼400 nm was determined as the optimal size in this study due to the high scattered peaks in the visible region of the solar spectrum (Figure S1b). The contents of ZrO_2_ and PTFE NPs were optimized for preparing NPs/NADES@PAAm/PVA in terms of solar reflectance (Table S2). The solar reflectance increased from 0.02 to 0.89 with increasing both ZrO_2_ and PTFE NPs from 0 to 100, while the solar reflectance leveled off when both ZrO_2_ and PTFE NPs exceeded 75%, and thus, 75 + 75% (ZrO_2_ + PTEF NPs) was regarded as the optimal content. Besides, the solar reflectance of NPs/NADES@PAAm/PVA decreased from 0.89 to 0.79 as the NADES concentration increased from 0 to 40%, while 10% NADES influenced insignificantly the solar reflectance, which was thus determined as the optimized concentration (Figure S2).

The SEM images showed that NPs are firmly embedded in the hydrogel (Figure 1c). In practical applications, NPs/NADES@PAAm/PVA does not contact the food, ensuring the food is without contamination. The FTIR spectrum shows that NPs/NADES@PAAm/PVA has strong emission bands at 1000–1280 and 1000–1260 cm^–1^, which are due to the vibrations of CF and CF_2_ of the PTFE and vibrations of C–O of PVA, and these absorbance bands are within the atmospheric window (770–1250 cm^–1^), indicating that NPs/NADES@PAAm/PVA tends to dissipate heat to outer space through atmospheric windows (Figure 1d). Overall, this study proposes a facile strategy for preparing NPs/NADES@PAAm/PVA to protect fruits from sunburn and provide a subambient temperature environment for fruit storage *via* combined effects of evaporative cooling and radiative cooling.

### 2.2 Passive Cooling Mechanism of NPs/NADES@PAAm/PVA

**Figure 2:**
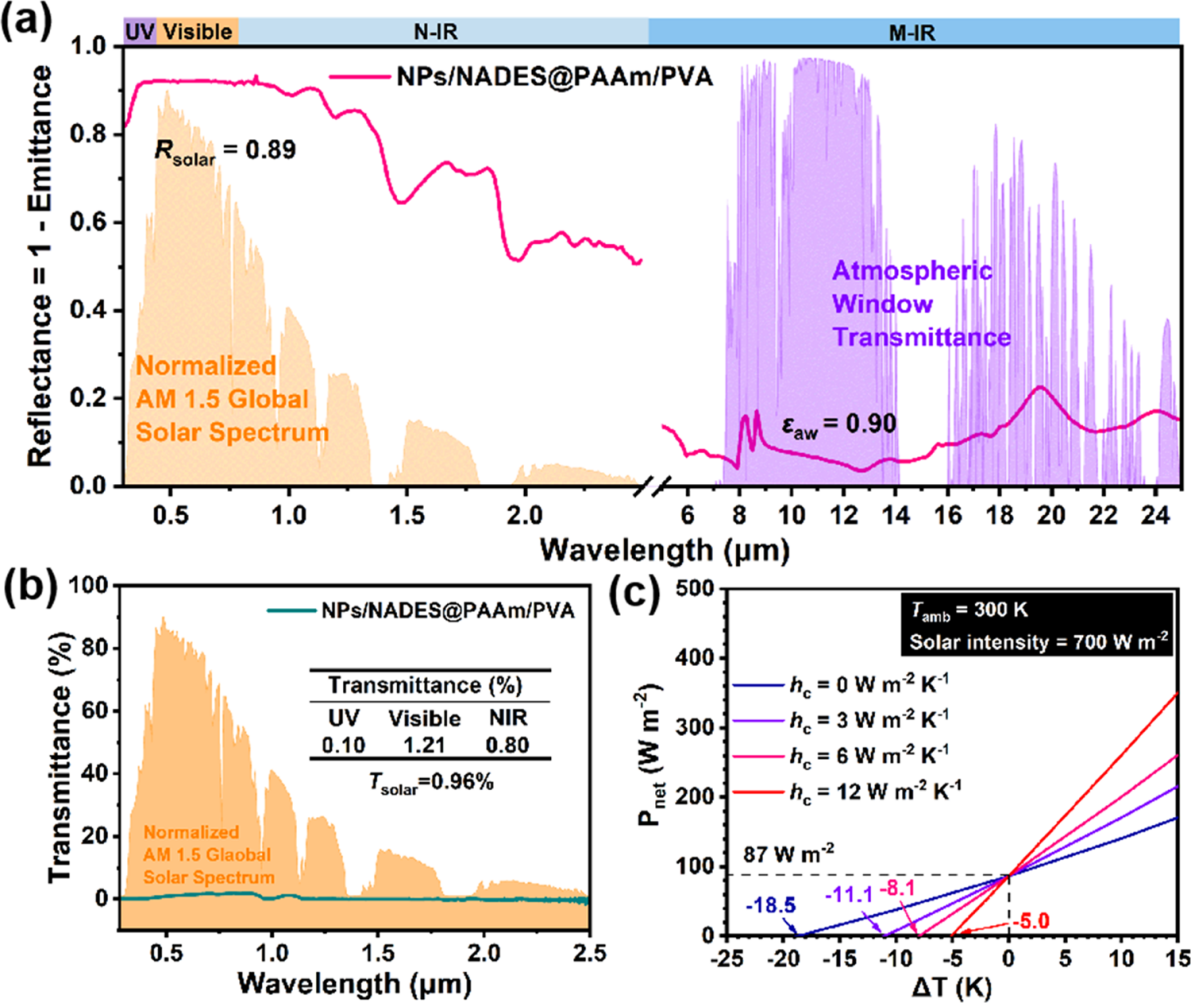
(a) Reflectance and emittance spectra of NPs/NADES@PAAm/PVA, including the normalized ASTM G173 Global solar spectrum and atmospheric transparency window for illustrating spectral selectivity. (b) Low solar transmittance of NPs/NADES@PAAm/PVA with the solar spectrum. The inset table shows the solar transmittance of NPs/NADES@PAAm/PVA in the different bands. (c) Theoretical cooling power of NPs/NADES@PAAm/PVA as a function of temperature difference Δ*T* = *T* – *T*_amb_ (*T*_amb_ = 300 K) with different heat transfer coefficients *h*_c_ = 0, 3, 6, and 12 W m^–2^ K^–1^, respectively.

Thermal radiation, convection, and conduction are the three main heat transfer modes. Excluding convection and conduction, an object can be cooled only when the quantity of heat released to an environment through radiation is greater than that of heat received. Besides, the atmosphere in the wavelength range of 8–13 μm has high transmittance, termed the atmospheric window, and NPs/NADES@PAAm/PVA exhibits high emittance (ε_aw_ = 0.90) in the atmospheric window, achieving a cooling effect by transferring heat to the low-temperature outer space (∼3 K) through the atmospheric window (Figure 2a). According to Plank’s law, the radiative intensity increases as the temperature of the material increases, and the total radiative powers of the blackbody (ε_aw_ = 1.00) increase from 268 to 606 W m^–2^ as the temperature increases from 277 to 333 K (Figure S3). As for NPs/NADES@PAAm/PVA (ε_aw_ = 0.90), the total radiative powers are in the range of 241–545 W m^–2^ for temperatures between 277 and 333 K. As shown in Figure S4, the theoretical cooling power of NPs/NADES@PAAm/PVA is evaluated according to the well-developed thermal balance model (Method S1). It is assumed that the temperature of NPs/NADES@PAAm/PVA is identical to the ambient temperature (*T*_amb_ = 300 K), and the corresponding thermal radiation power (*P*_rad_) is calculated as 347 W m^–2^. Compared with thermal radiation, solar radiation absorption of a material is a more critical factor in achieving a radiative cooling effect during the daytime. Although the solar radiation intensity is detected as about 700 W m^–2^, due to the high solar reflectance of NPs/NADES@PAAm/PVA (*R*_sol_ = 0.89), the absorbed solar power (*P*_sol_) is 77 W m^–2^. Furthermore, the absorbed atmospheric radiation power (*P*_atm_) of 183 W m^–2^ is almost half of *P*_rad_, demonstrating that the radiative cooling effect is susceptible to atmospheric window transmittance, which is affected by environmental conditions, including cloud cover, humidity, and air pollutants.^29^ When the effects of heat convection and conduction are excluded, the theoretical net radiative cooling (*P*_net-rad_) is 87 W m^–2^, indicating that NPs/NADES@PAAm/PVA can achieve radiative cooling. Besides, the solar transmittance of NPs/NADES@PAAm/PVA is nearly light-proof (*T*_solar_ = 1.0%), and the ultraviolet-proof is almost perfect (*T*_solar-uv_ = 0.1%), suggesting that NPs/NADES@PAAm/PVA can be an effective sun-proof material (Figure 2b). On the contrary, as shown in Figure S5, the solar reflectance and emittance at the atmospheric window of PAAm/PVA are 0.23 and 0.03, respectively. The *P*_net-rad_ of PAAm/PVA is calculated to be −17 W m^–2^, demonstrating that PAAm/PVA fails to achieve radiative cooling. Besides, the solar transmittance of PAAm/PVA is 73.5%, indicating that PAAm/PVA cannot protect the fruit from solar radiation by shielding sunlight.

Apart from thermal radiation, heat convection and conduction can largely affect cooling performance, and the influences of convection and conduction on cooling performance can be quantified using the combined conductive and convective heat transfer coefficient (*h*_c_), which has a linear correlation with wind velocity.^30^ As shown in Figure 2c, compared with ambient temperature (*T*_amb_ = 300 K), the relative maximum cooling temperatures (Δ*T*) of NPs/NADES@PAAm/PVA are −18.5, −11.1, −8.1, and −5.0 °C as the *h*_c_ values are 0, 3, 6, and 12 W m^–2^ K^–1^, respectively, suggesting that a high *h*_c_ value is adverse to the subambient temperature reduction of NPs/NADES@PAAm/PVA, while these nonradiation heat effects can be reduced by an infrared-transparent cover shield or vacuum chamber.^31,32^ Apart from radiative cooling, evaporative cooling can dissipate a large amount of heat and thus maintain the samples at a relatively low temperature under solar radiation due to the high enthalpy of water vaporization, which is largely influenced by the temperature and evaporative area.^21^ Therefore, NPs/NADES@PAAm/PVA can realize the sun-proof and cooling functions under sunlight based on the high reflectance in the solar spectrum, high emittance in the atmospheric window, and adequate water evaporation.

### 2.3 Mechanical and Swelling Properties

**Figure 3:**
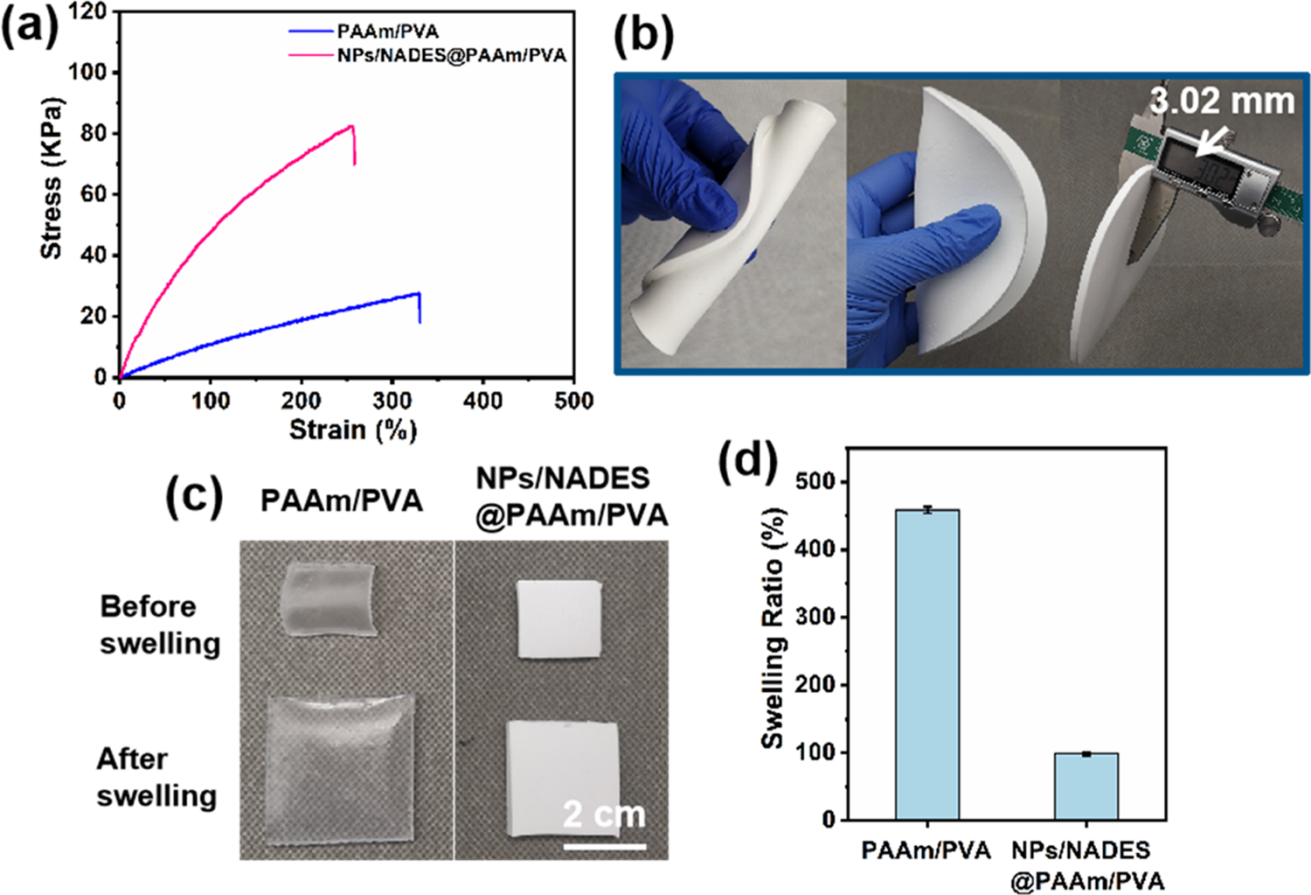
(a) Stress–strain curves of PAAm/PVA and NPs/NADES@PAAm/PVA. (b) Photographs showing the performance of NPs/NADES@PAAm/PVA with ∼3 mm thickness, including rolling and folding. (c) Photographs of PAAm/PVA and NPs/NADES@PAAm/PVA before swelling and the corresponding samples after 24 h swelling. (d) Swelling ratios of PAAm/PVA and NPs/NADES@PAAm/PVA.

Apart from sun-proof and cooling functions, the mechanical strength of the materials plays an essential role in practical applications, mainly including the reduction in the mechanical strength of hydrogel after water absorption. For many hydrogels, the typical swelling-weakening phenomenon occurs due to the network dilution after swelling, suffering from a sharp decrease in mechanical strength.^33−35^ Hence, the mechanical properties of hydrogels with different treatments were investigated, and the results are shown in Figure 3a. The tensile stress and elongation at the break of PAAm/PVA were 27.5 kPa and 330.0%, respectively. After incorporating NPs and NADES into the PAAm/PVA, the tensile stress of NPs/NADES@PAAm/PVA was significantly enhanced to 82.5 kPa, while its elongation was decreased to 256.2%. A possible mechanism for enhancing tensile stress is that the NPs can serve as pseudo crosslinkers attributed to the non-covalent interactions between NPs and PAAm/PVA chains, thus increasing the cross-linking degree of the hydrogel network.^36,37^ As shown in Table S3, after incorporating NADES into the PAAm/PVA, the tensile stresses of NADES@PAAm/PVA decreased to 15.0 kPa, while the elongation increased to 446.1%, respectively. The decrease of tensile stress can be attributed to the enhanced hydrophilicity provided by the NADES system, resulting in better-swelling properties and thus a stronger swelling-weakening effect, and the reason for the increased elongation of the hydrogel can be due to more hydroxy groups from NADES for producing stronger hydrogen bond supramolecular networks.^24^ Furthermore, NPs/NADES@PAAm/PVA has excellent flexibility and can withstand rolling or folding, as shown in Figure 3b. Besides, the swelling property is the most crucial property of hydrogels. When a hydrogel is immersed in water, the hydrogel absorbs and stores water, which can thus be adopted as a liquid–gas phase-change material to realize the cooling effect, resulting in a larger cooling capacity than radiative cooling alone. Hence, the swelling properties of the hydrogels with different treatments were determined, as shown in Figure 3c,d. The photograph shows that the expansion of the hydrogel obviously decreased after the NP and NADES incorporation, and the swelling ratio of the PAAm/PVA (458.8%) was approximately fivefold that of NPs/NADES@PAAm/PVA (98.8%) after 24 h water swelling. These results indicate that the PAAm/PVA possesses good water accessibility due to numerous hydrophilic groups, while the NPs reduce the porosity of the hydrogel, leading to the decreasing water-absorbing capacity. Nevertheless, since the NADES can improve the hydrophilicity of the system, NADES@PAAm/PVA (473.2%) and NPs/NADES@PAAm/PVA (98.8%) exhibited larger swelling ratios compared with PAAm/PVA (458.8%) and NPs@PAAm/PVA (89.3%), respectively (Table S3). These swelling behaviors of hydrogel provide an opportunity to combine evaporative cooling with radiative cooling, achieving better cooling performance. Overall, NPs/NADES@PAAm/PVA exhibits robust mechanical properties, excellent flexibility, and good swelling behavior, laying a solid foundation for practice applications.

### 2.4 NPs/NADES@PAAm/PVA for Sunburn Resistance and Temperature Stabilization

**Figure 4:**
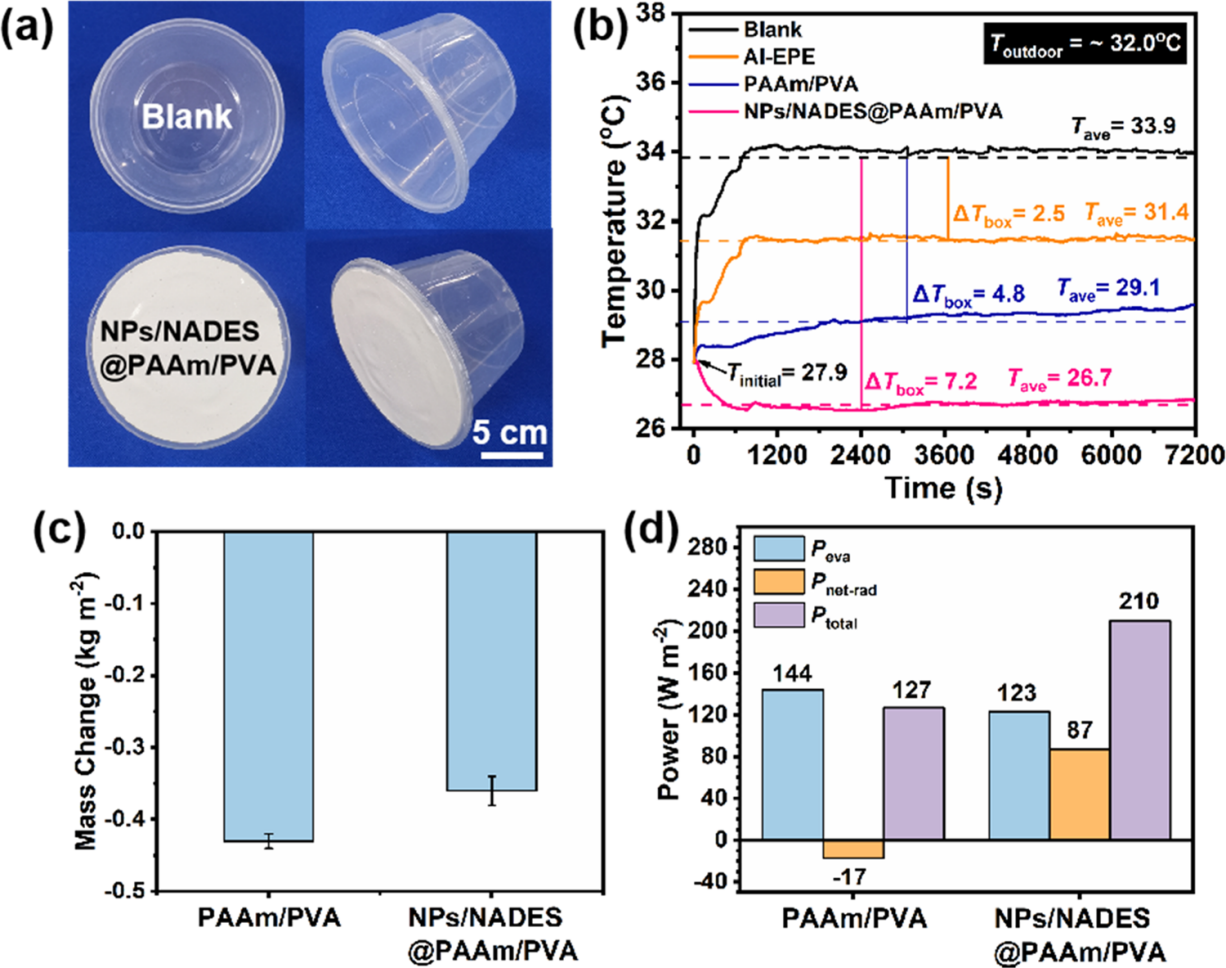
(a) Photographs showing a food preservation box without treatment (blank) and a food preservation box covered by NPs/NADES@PAAm/PVA. (b) Temperature changes inside the food preservation box with different treatments under ∼700 W m^–2^ sunlight from 11:00 to 13:00 in April 2022 in Guangzhou, China. (c) Mass changes of PAAm/PVA and NPs/NADES@PAAm/PVA under ∼700 W m^–2^ sunlight after 2 h. (d) Theoretical evaporative, radiative, and total cooling powers of PAAm/PVA and NPs/NADES@PAAm/PVA.

To verify the sun-proof and cooling functions of NPs/NADES@PAAm/PVA, several food preservation boxes were firmly covered by PAAm/PVA, Al-EPE, or NPs/NADES@PAAm/PVA, respectively, using water-proof adhesive and set under the sunlight to determine the cooling effect (Figure 4a). As shown in Figure 4b, after sunlight exposure, the blank temperature sharply increased from 27.9 to 34.1 °C during the first 750 s and then levelled off, indicating that solar radiation readily heated up the box. Compared with the blank, the box treated with Al-EPE obtained a lower equilibrium temperature (from 27.9 to 31.5 °C) during the first 750 s due to the high solar reflectance. Meanwhile, the temperature of the box treated with PAAm/PVA gently increased from 27.9 to 28.4 °C during the first 750 s. The results suggest that the PAAm/PVA effectively inhibits the temperature increase compared with the Al-EPE because PAAm/PVA absorbs a large amount of heat during water evaporation. Noteworthily, the temperature of the box treated with NPs/NADES@PAAm/PVA decreased from 27.9 to 26.5 °C during the first 750 s and then levelled off. This could be due to the high solar reflectance and the combined effect of evaporative and radiative cooling of NPs/NADES@PAAm/PVA, reducing the solar radiation reception and providing a cold source for the box. After 2 h of sun exposure, the blank reached the highest average temperature of 33.9 °C inside the box, which was higher than the average outdoor temperature (*T*_outdoor_ = ∼32 °C) due to the absorption of solar radiation, while the average temperature of the box treated with Al-EPE as a positive control group exhibited a lower value of 31.4 °C attributed to the high solar reflectance. Noteworthily, compared with the blank, the average temperature difference inside the box (Δ*T*_box_) treated with PAAm/PVA was 4.8 °C, thanks to evaporative cooling. Combining radiative cooling with evaporative cooling, the average temperature decrease inside the box treated with NPs/NADES@PAAm/PVA was the highest, achieving a Δ*T*_box_ of 7.2 °C, indicating that NPs/NADES@PAAm/PVA can effectively cool the food preservation box under the sunlight. Furthermore, mass changes in PAAm/PVA and NPs/NADES@PAAm/PVA were determined, and the water loss in PAAm/PVA was higher than that in NPs/NADES@PAAm/PVA, indicating that water evaporation in PAAm/PVA is more effective, which can be ascribed to the higher temperature of the hydrogel (Figure 4c). Correspondingly, the evaporative cooling power (*P*_eva_) of PAAm/PVA (144 W m^–2^) was higher than that of NPs/NADES@PAAm/PVA (123 W m^–2^). Due to the negative value of radiative cooling power (*P*_net-rad_ = −17 W m^–2^), the theoretical total cooling power (*P*_total_) of PAAm/PVA was calculated to be 127 W m^–2^. This result indicates that the higher temperature of PAAm/PVA compared with that of NPs/NADES@PAAm/PVA could be attributed to the lower *P*_net-rad_. Noteworthily, the *P*_total_ of NPs/NADES@PAAm/PVA was calculated to be 210 W m^–2^, including 123 W m^–2^ by *P*_eva_ and 87 W m^–2^ by *P*_net-rad_, explaining the superior cooling performance of NPs/NADES@PAAm/PVA.

Furthermore, to verify the reliability of NPs/NADES@PAAm/PVA during the daytime, the outdoor tests for cooling performance were conducted from 9:20 to 17:20 on a sunny day in August 2022 in Guangzhou, China (Figure S6a). As shown in Figure S6b, the temperature of the food preservation box treated with NPs/NADES@PAAm/PVA decreased from 28.0 to 26.7 °C within ∼5 h (from 9:30 to 14:20), and its maximum temperature decrease (Δ*T*_max_) achieved 5.8 °C compared with ambient temperature, indicating that NPs/NADES@PAAm/PVA can effectively stabilize the temperature of the box from morning to afternoon. This could be attributed to the combined effect of evaporative and radiative cooling of NPs/NADES@PAAm/PVA. Strong solar radiation (∼700 W m^–2^) and low relative humidity (∼31%) at noon are conducive to water evaporation, resulting in effective evaporative cooling (Figure S6d). However, the temperature dramatically increased to 29.7 °C from 14:20 to 15:15, which could be due to the water loss leading to the reduction of evaporative cooling. The water retention decreased from 50.0 to 2.8%, and the remaining water could be regarded as ineffective water (Figure S6c). This could be due to the hydrophilicity of the material, restraining water evaporation. Therefore, after the water runs out, NPs/NADES@PAAm/PVA needs to be rehydrated by immersing in the water to restore evaporative cooling. Due to radiative cooling, the temperature of the box was still lower than the ambient temperature from 15:15 to 17:20, and its Δ*T*_max_ was 1.8 °C compared with the ambient temperature. All in all, NPs/NADES@PAAm/PVA is reliable in stabilizing the temperature of the box for ∼5 h under sunlight through the combined effect of evaporative and radiative cooling.

**Figure 5:**
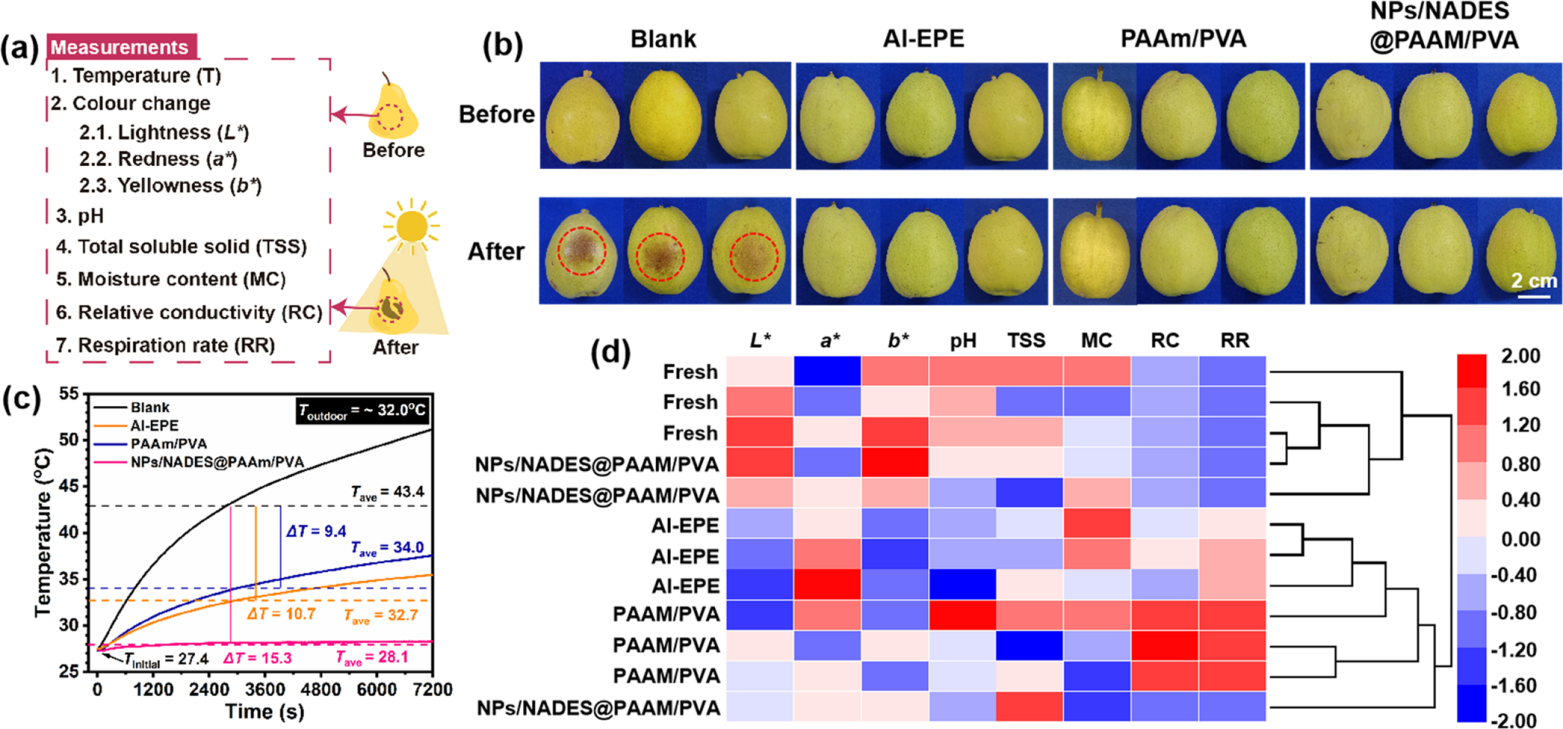
(a) Schematic diagram showing the characterization of *P. sinkiangensis* (PS). (b) Photographs showing PS with different treatments after ∼700 W m^–2^ sun exposure. (c) Temperature changes in PS with different treatments during sun exposure from 11:00 to 13:00 in April 2022 in Guangzhou, China. (d) Heatmap combined with the dendrogram of cluster analysis obtained based on the determined quality attributes of PS with different treatments after sun exposure.

To go further, fruit preservation experiments were performed, and the quality attributes of pears (*Pyrus sinkiangensis*) stored in the food preservation box after 2 h sunlight exposure were analyzed. The discoloration in the peel involving sunburn was associated with increased concentrations of quercetin glycosides and carotenoids and decreased concentrations of anthocyanins and chlorophylls, leading to the quality deterioration of pears.^38^ As shown in Figure 5b, skin browning only occurred on sun-exposed sections of the pears, while there were insignificant changes in pear skin after different treatments. To quantify the color changes of the pears, the values of L* (lightness), a* (redness), and b* (yellowness) were measured by a colorimeter. As displayed in Table 1, the L* and b* decreased and a* increased in the pear sunburn part after sun exposure, while the color of the pears with different treatments changed insignificantly. As shown in Figure 5c, the temperature changes in pear with different treatments were determined, and the results showed that the average temperature (*T*_ave_) of the blank was 43.4 °C, while the other treatment groups effectively inhibited the temperature increment. Particularly, compared with the blank, the NPs/NADES@PAAm/PVA treatment exhibited the highest relative average temperature decrease (Δ*T*) of 15.3 °C, followed by Al-EPE treatment (10.7 °C) and PAAm/PVA treatment (9.4 °C), indicating that solar reflectance is more important than evaporative cooling for temperature reduction due to the high solar radiation intensity, while combining solar reflectance with radiative and evaporative cooling can achieve superior temperature stabilization performance. Furthermore, the comparison of the pear quality attributes with different treatments before and after sun exposure is summarized in Table 1, including pH, total soluble solids (TSS), moisture content (MC), relative conductivity (RC), and respiration rate (RR). RR involving the metabolic activity of fruit plays a vital role in shelf-life, which can be regulated by storage temperatures. Since the temperature of the blank increased from 27.3 to 51.2 °C after sun exposure, the RR of the pear increased from 25.24 to 58.42 mg kg^–1^ h^–1^, while the RR of the pear treated with NPs/NADES@PAAm/PVA exhibited the lowest RR of 25.68 mg kg^–1^ h^–1^ due to better temperature stabilization compared with the other groups. Besides, the RC of pear with different treatments after sun exposure was determined as blank > PAAm/PVA > Al-EPE > NPs/NADES@PAAm/PVA (43.59 > 30.41 > 20.82 > 18.25%), which might be due to the cell membrane damage caused by solar radiation involving the increased permeability of the cell membrane and thus electrolyte leakage from the cell.^39^ Besides, higher temperatures can cause higher metabolic activities of fruits, promoting faster macromolecular decompositions into micromolecules and their leaking out of the cell,^40^ while NPs/NADES@PAAm/PVA with the sun-proof and cooling functions can effectively maintain the cell membrane integrity and nutrients. The pH of the blank increased after sun exposure, which can be attributed to the loss of organic acids.^5^ The TSS of the blank decreased after sun exposure, which can be ascribed to the loss of saccharide.^5^ In contrast, the pH and TSS of NPs/NADES@PAAm/PVA changed insignificantly. Apart from pears, the preservation of Fuji apples was also investigated, as shown in Figure S8. Similarly, the apples treated with NPs/NADES@PAAm/PVA exhibited the highest temperature decrease and the lowest RR for preservation under sunlight.

**Table 1:** Quality Comparison of *P. sinkiangensis* with Different Treatments after Sun Exposure^a^

		color parameters							
treatments	sun exposure	L*	a*	b*	pH	TSS (%)	MC (%)	RC (%)	RR (mg kg^–1^ h^–1^)
blank	before	62.29 ± 0.89^Aa^	–3.37 ± 0.93^Aa^	40.65 ± 0.82^Aa^	5.88 ± 0.01^Aa^	12.20 ± 0.29^Aa^	84.51 ± 0.27^Aa^	18.91 ± 0.48^Aa^	25.24 ± 0.10^Aa^
	after	37.41 ± 0.68^Bc^	6.35 ± 0.68^Ba^	19.44 ± 0.69^Bc^	6.09 ± 0.02^Ba^	10.50 ± 0.08^Bc^	83.83 ± 0.07^Aa^	43.59 ± 0.77^Ba^	58.42 ± 1.14^Ba^
Al-EPE	before	62.28 ± 1.05^Aa^	–2.20 ± 0.86^Aa^	39.37 ± 0.59^Aa^	5.79 ± 0.08^Aa^	12.50 ± 0.22^Aa^	83.97 ± 0.18^Aa^	17.97 ± 0.61^Aa^	25.49 ± 0.21^Aa^
	after	60.25 ± 0.84^Ab^	–1.12 ± 0.76^Ab^	36.97 ± 0.54^Ab^	5.70 ± 0.05^Ab^	11.27 ± 0.12^Bb^	84.74 ± 0.20^Aa^	20.82 ± 1.72^Bc^	32.62 ± 0.74^Bc^
PAAm/PVA	before	62.85 ± 0.96^Aa^	–3.40 ± 0.74^Aa^	40.32 ± 0.63^Aa^	5.82 ± 0.17^Aa^	12.40 ± 0.36^Aa^	83.96 ± 0.30^Aa^	18.13 ± 0.42^Aa^	25.44 ± 0.17^Aa^
	after	60.26 ± 1.07^Ab^	–2.32 ± 0.96^Ab^	38.16 ± 0.82^Ab^	5.84 ± 0.10^Ab^	11.07 ± 0.34^Bb^	84.40 ± 0.30^Aa^	30.41 ± 0.43^Bb^	36.09 ± 0.24^Bb^
NPs/NADES@PAAm/PVA	before	62.78 ± 1.64^Aa^	–2.45 ± 0.67^Aa^	40.95 ± 0.99^Aa^	5.89 ± 0.05^Aa^	12.37 ± 0.45^Aa^	84.30 ± 0.26^Aa^	17.40 ± 0.74^Aa^	25.61 ± 0.17^Aa^
	after	62.63 ± 1.37^Aa^	–2.60 ± 0.72^Ab^	40.94 ± 1.06^Aa^	5.86 ± 0.03^Ab^	12.17 ± 0.37^Aa^	84.40 ± 0.23^Aa^	18.25 ± 0.77^Ad^	25.68 ± 0.14^Ad^

a Three replicates were tested for each measurement, and data were expressed as means ± standard deviations. Significance labels containing different uppercase letters are different by Student’s *t*-test (*p* < 0.05) in the same group, and significance labels containing the same lowercase letter are not different by Duncan’s test (*p* < 0.05) in the same item. L*, lightness; a*, redness; b*, yellowness; TSS, total soluble solids; MC, moisture content; RC, relative conductivity; RR, respiration rate.

Overall, combining radiative and evaporative cooling, NPs/NADES@PAAm/PVA shows a great potential to protect the fruits such as citrus, pomegranate, lemon, mango, grape, orange, pineapple, *etc.*, which often suffer from sunburn and the lowering of the storage quality due to high ambient temperatures.^41−44^

### 2.5 Multivariate Data Analysis

**Figure 6:**
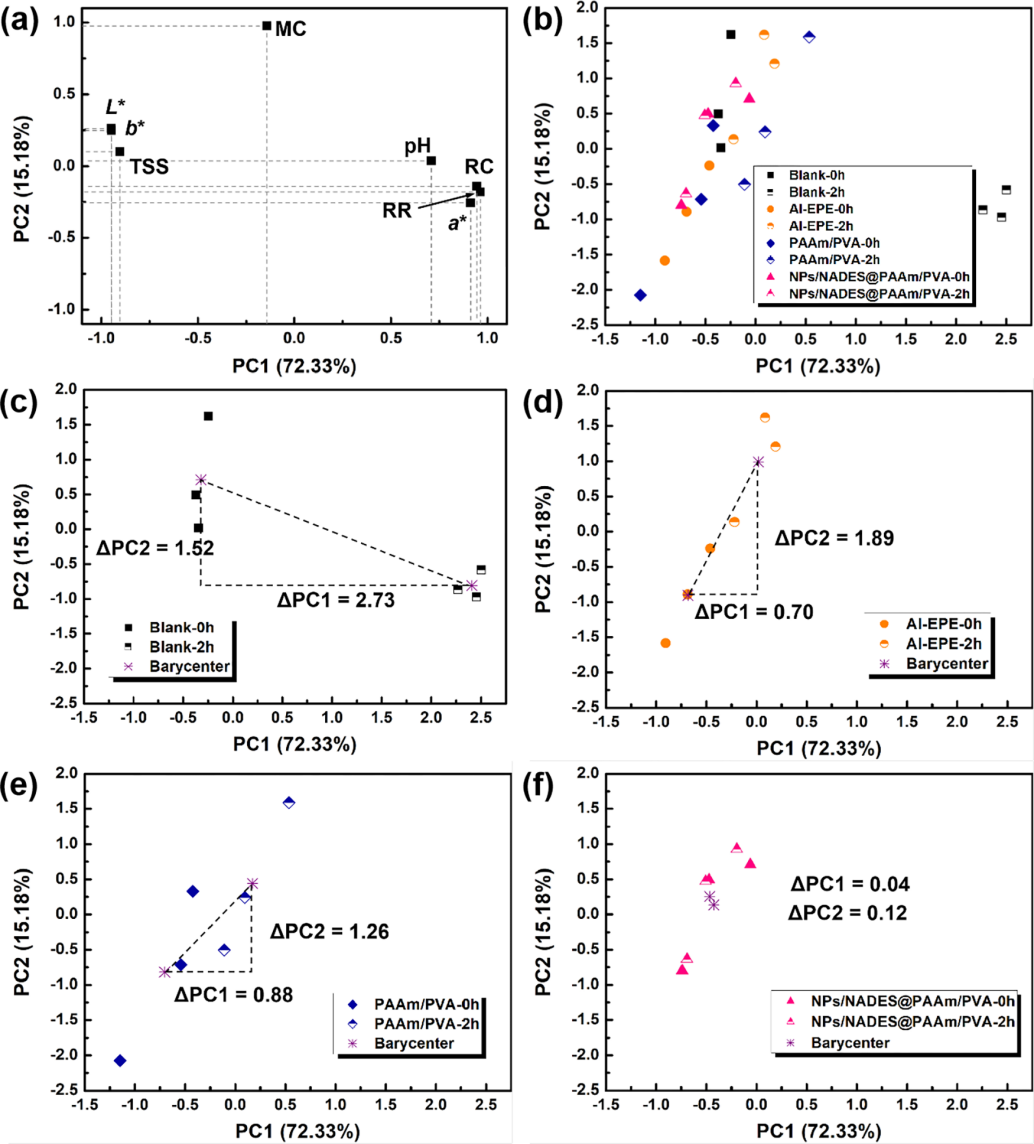
Principal component analysis (PCA) of the *P. sinkiangensis* (PS) quality with different treatments after 0 and 2 h sun exposure. Three replicates were tested for each measurement. (a) Loading variable plot of PCA. (b) Comprehensive PCA score plot that compares fresh PS and PS after 2 h sun exposure with different treatments. Individual PCA score plots of (c) blank, (d) Al-EPE, (e) PAAm/PVA, and (f) NPs/NADES@PAAm/PVA for evaluating the quality change of PS after 2 h sun exposure. ΔPC1 and ΔPC2 represent the migration distance of the data barycenter in PC1 and PC2, respectively.

As an unsupervised pattern recognition method, principal component analysis (PCA) can obtain a few comprehensive indicators to represent multiple indicators by means of dimensionality reduction.^45^ A coordinate system is established by defining the first two principal components that possess an eigenvalue greater than 1.0. To better illustrate and understand the quality variation of pear after sun exposure, PCA involving fresh pear (0 h) and pear after sun exposure (2 h) was performed. Figure 6a displays the loading information of PCA involving the tested variables. The first principal component (PC1) positively correlated with a*, RH, and RR and negatively correlated with L*, b*, and TSS, accounting for 72.33% of the total variance, while the second principal component (PC2) positively correlated with MC, accounting for 15.18% of the total variance. PC1 and PC2 together explained 87.51% of the total variance, suggesting that the most significant quality change of pear after sun exposure was contributed by RR, RC, and color change. Figure 6b displays the PCA score plot comparing fresh pear (0 h) and pear after sun exposure (2 h) with different treatments, which was decomposed into four individual score plots corresponding to four treatments (Figure 6c–f). After sun exposure, the blank (Figure 6c) exhibited the greatest migration distances in both PC1 and PC2 (ΔPC1 = 2.73, ΔPC2 = 1.52), while NPs/NADES@PAAm/PVA (Figure 6f) displayed the lowest migration distances in both PC1 and PC2 (ΔPC1 = 0.12, ΔPC2 = 0.04), and since greater values of ΔPC1 and ΔPC2 suggested a more severe quality loss, NPs/NADES@PAAm/PVA endowed the pear with the best quality stabilization after sun exposure. Unlike PCA, hierarchical cluster analysis (HCA) can be used to treat the high-dimensional data matrix by ignoring the category of samples.^46^ The HCA consisting of a heatmap of variable values and a dendrogram of the clustering result is shown in Figure 5c, and four groups of the pear were mainly clustered into two big groups: fresh pear and pear treated with NPs/NADES@PAAm/PVA after sun exposure in the first group, while pear treated with PAAm/PVA or Al-EPE after sun exposure in the second group. Although there was an NPs/NADES@PAAm/PVA group clustered in the second group, it was observed that the data of the Al-EPE and PAAm/PVA groups were merged first, and the data of the NPs/NADES@PAAm/PVA group were finally integrated into the same cluster. The heatmap shows that Al-EPE and PAAm/PVA groups presented significantly higher RC and RR and relatively lower L* and b*, while the NPs/NADES@PAAm/PVA group shared more similarities with the fresh pear. Overall, the predominant results of PCA and HCA provided the same conclusion that pears treated with NPs/NADES@PAAm/PVA exhibited the most similar quality to the fresh ones, announcing its promising prospects for fruit preservation under solar radiation.

## 3 Conclusions

A nanocomposite hydrogel is developed for fruit preservation under solar radiation. The NPs endow the hydrogel with high solar reflectance, atmospheric window emittance, and robust mechanical properties, while the NADES enhances the hydrogel’s stretchability and moisture sorption capacity. After water absorption, NPs/NADES@PAAm/PVA exhibits an excellent passive cooling effect by associating radiative and evaporative cooling. The results show that the pear treated with NPs/NADES@PAAm/PVA achieve the highest average temperature decrease and the most similar quality attributes to the fresh one after sun exposure compared with the other treatments, suggesting that NPs/NADES@PAAm/PVA can effectively prevent the fruit from sunburn and high-temperature stress, and thus, the current study provide an alternative avenue of sun-proof and temperature stabilization in fruit preservation under solar radiation.

## 4 Materials and Methods

### 4.1 Materials

d-Sorbitol (Sor, C_6_H_14_O_6_), l-proline (Pro, C_5_H_9_NO_2_), poly(vinyl alcohol) 1799 (PVA), acrylamide (AAm, monomer), *N*,*N*′-methylenebisacrylamide (MBA, cross-linking agent), ammonium persulphate (APS, initiator), and *N*,*N*,*N*′,*N*′-tetramethylethylenediaminee (TMEDA, accelerant) were purchased from Aladdin Reagent Co., Ltd. (Shanghai, China). Zirconium dioxide nanoparticles (ZrO_2_ NPs, 400 nm) and poly(tetrafluoroethylene) nanoparticles (PTFE NPs, 400 nm) were acquired from Shanghai Yaoyi Alloy Material Co., Ltd. (Shanghai, China). All of the chemicals were of analytical grade. The pears (*P. sinkiangensis*) and Fuji apples were purchased from a local market (Guangzhou, China).

### 4.2 Preparation of Natural Deep Eutectic Solvent (NADES)

NADES was prepared according to our previous method.^47^ Briefly, Sor and Pro were mixed in a 1:1 molar ratio, and the mixture was stirred at 50 °C and 100 rpm using a magnetic stirrer (C-MAG HS10, IKA GmbH, Staufen, Germany) until a transparent and viscous liquid was obtained.

### 4.3 Preparation of NPs/NADES@PAAm/PVA

NPs/NADES@PAAm/PVA was prepared by one-pot liquid casting and free radical polymerization, comprising high concentrations of ZrO_2_ and PTFE NPs. Figure 1a shows the schematic diagram for the preparation process of nanoparticle composite hydrogel added with NADES. First, PVA was dissolved in deionized water at 90 °C and 500 rpm using a magnetic stirrer to obtain a 10 wt % homogeneous PVA solution. Afterward, about 50 wt % PVA solution, 40 wt % AAm, 0.12 wt % MBA, and 0.34 wt % APS were mixed as mother liquor. Then, four sols for different types of hydrogels were prepared by mixing ZrO_2_ NPs, PTFE NPs, and NADES with the mother liquor in a certain mass ratio (Table S1), and the mixture was homogenized (FJ200, Shanghai Specimen Model Factory, Shanghai, China) until well-dispersed sols were obtained. After 0.6% TMEDA (w/w, TMEDA/ mother liquor) was mixed with the sol completely using a glass rod in half a minute, the hybrid sol was poured into a custom-made mold and the reaction was carried out at room temperature of 25 °C for 5 min to obtain the four hydrogels with different treatments. Finally, the swollen samples were obtained by peeling off the mold and immersed in water at 25 °C for 24 h.

### 4.4 Characterization of Fabricated Hydrogels

The thickness of the samples, which was dependent on the volume of the hybrid sol, was measured using an electronic vernier caliper (MNT-300, Meinaite Inc., Shanghai, China). Fourier transform infrared (FTIR) spectra of the samples were obtained using an FTIR spectrometer (Tensor 27, Bruker Inc., Karlsruhe, Germany) in an attenuated total refraction module. Scanning electron microscopy (SEM) images were taken by a field-emission scanning electron microscope (Merlin, Carl Zeiss NTS GmbH, Oberkochen, Germany).

The spectral reflectance and transmittance in ultraviolet, visible, and near-infrared wavelength ranges (0.3–2.5 μm) were characterized by an ultraviolet–visible–near-infrared (UV–vis–NIR) spectrophotometer (PerkinElmer Lambda 750S, PerkinElmer Inc., Massachusetts) with a poly(tetrafluoroethylene) integrating sphere. The solar reflectance (*R*_sol_) of NPs/NADES@PAAm/PVA was defined as
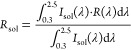
1where λ is the wavelength, *I*_sol_(λ) is the normalized ASTM G173 global solar intensity spectrum, and *R*(λ) is the spectral reflectance of NPs/NADES@PAAm/PVA.

The spectral reflectance ρ(λ) and transmittance τ(λ) in the mid-infrared wavelength ranges (8–13 μm) were characterized using an FTIR spectrometer (Nicolet iS50, Thermo Fisher Scientific Inc., Massachusetts) with a gold integrating sphere. As the emittance (ε(λ)) and absorptivity of any object are identical according to Kirchhoff’s law, ε(λ) could be calculated as ε(λ) = 1 – ρ(λ) – τ(λ). The emittance at the atmospheric window (ε_aw_) of NPs/NADES@PAAm/PVA was defined as
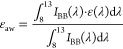
2where ε(λ) is the emittance of NPs/NADES@PAAm/PVA,  is the radiance of a blackbody at temperature *T* = 300 K, *h* is the Planck constant, *K*_B_ is the Boltzmann constant, and *c* is the speed of light.

### 4.5 Mechanical Strength

The tensile properties of hydrogels were measured using a servo material testing machine (HZ-1007E, Li Xian Instrument Co., Ltd., Dongguan, China) at room temperature (25 °C) by setting the stretch speed at 10 mm min^–1^. Hydrogels were first immersed in water at 25 °C for 24 h, and then, the swollen hydrogels were cut into dumbbell shapes. The specimens had a width of 10 mm and a thickness of 5 mm. The gauge length between the clamps was 50 mm.

### 4.6 Swelling Behavior

The hydrogels were first dried at 70 °C for 12 h and then cut into blocks of a size of about 2 mm × 2 mm × 2 mm, and the blocks were then immersed in deionized water at room temperature (25 °C) for 24 h. The swelling ratios of the hydrogels could be calculated according to the following equation
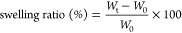
3

where *W_t_* and *W*_0_ are the weight of the swollen hydrogel at time *t* and the original weight of the hydrogel, respectively.

### 4.7 Mass Changes and Theoretical Evaporative Cooling Power of the Hydrogel

The mass changes could be calculated according to eq 4
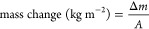
4where Δ*m* and *A* are the weight loss and area of the hydrogel, respectively, and the theoretical evaporative cooling power (*P*_eva_^theo^) can be calculated according to eq 5
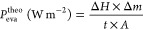
5where Δ*H* is the enthalpy of water vaporization (2435 J g^–1^) at a temperature of 28 °C, *t* is the evaporation time, and Δ*m* and *A* are the weight loss and area of the hydrogel, respectively.

### 4.8 Characterization of Pear (*P. sinkiangensis*) Quality Attributes

The pears were stored in a food preservation box (15 cm × 15 cm × 8.5 cm) to compare the quality of the pears with different treatments after sun exposure. The lid of the food preservation box was covered by aluminum foil with expanded polyethene (Al-EPE), PAAm/PVA, or NPs/NADES@PAAm/PVA using water-proof adhesive (Ergo5210, Kisling Co., Ltd., Wetzikon, Switzerland). Then, the box was put into a custom-made container. In the container, polystyrene foam was employed for thermal insulation, which was covered by aluminum foil to reflect sunlight irradiation, and a windshield was applied to reduce heat convection (Figure S7). After 2 h of sun exposure, the quality attributes of pears were analyzed. The values of L* (lightness), a* (redness), and b* (yellowness) in the sunburn part of the pears were measured by a colorimeter (NS820, Shenzhen Sanenshi Technology Co., Ltd., Shenzhen, China). From the pears in the sunburn part, 5 mL of juice was squeezed out, and the pH value was measured with a pH meter (PHSJ-4F, Shanghai Yidian Scientific Instrument Co., Ltd., Shanghai, China). About 0.3 mL of juice was applied to determine the total soluble solids (TSS) using a refractometer (PAL-1, ATAGO Co., Ltd., Tokyo, Japan). About 0.1 g of pulp from the pears in the sunburn part was sampled, and its moisture content (MC) was measured by a moisture meter (XY-100MW-T, Shanghai Jiazun Instrument Equipment Co., Ltd., Shanghai, China). About 0.5 g of pulp from the pears in the sunburn part was sampled and placed in a test tube with 10 mL of deionized water, and the mixture was homogenized at 5000 rpm for 5 min. The homogenate was applied to measure the electrolytic conductivity (EC) by a conductivity meter (DDS-307A, INESA Analytical Instrument Co., Ltd., Shanghai, China). After that, the homogenate was kept in a boiling water bath (HH-2, Changzhou Aohua Instrument Ltd., Changzhou, China) for 15 min and then cooled to room temperature (28 ± 0.5 °C), and EC was recorded. The relative conductivity (RC) was calculated according to eq 6
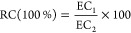
6where EC_1_ and EC_2_ represent the EC (S m^–1^) before and after boiling treatment, respectively. A pear (120 ± 10 g) was enclosed in a 500 mL glass jar that was connected to a breath meter (3051H, Zhejiang TOP Cloud-Agri Technology Co., Ltd., Zhejiang, China), and the respiration rate (RR) was evaluated by a breath meter and expressed in mg kg^–1^ h^–1^. The real-time temperatures of the samples were monitored at an interval of 2 s using T-type thermocouples (5SRTC-TT-T-30–36, Omega Engineering Inc., Norwalk) connected to a data logger (TC-08, OMEGA Engineering Inc., Norwalk). The solar power was monitored by an irradiatometer (CEL-FZ-A, Ceaulight Technology Co., Ltd., Beijing, China). The relative humidity was monitored by a hygrometer (COS-04, Shandong Renke Control Technology Co., Ltd., Shangdong, China).

### 4.9 Statistical Analysis

Three replicates for each sample were tested unless stated otherwise, and the data were expressed as means ± standard deviations. One-way variance analysis using Duncan’s test at a significant level of *p* < 0.05 was performed using SPSS 23.0 (SPSS Inc., Chicago), and multivariate data analyses, including principal component analysis (PCA) and hierarchical cluster analysis (HCA), were also performed using SPSS.

## Supporting Information Available

Details about the theoretical cooling power and water retention calculations, detailed information for preparing different hydrogels, optimization of PTFE NPs and ZrO_2_ NPs contents, mechanical and swelling properties of different hydrogels, photos of hydrogel precursors, scattering coefficients by ZrO_2_ NPs with various sizes, optimization of NADES concentrations, radiative spectra of a blackbody, power densities of NPs/NADES@PAAm/PVA, spectral characteristics and power densities of PAAm/PVA, outdoor tests for cooling performance of NPs/NADES@PAAm/PVA, custom-made device for real-time temperature determination, and outdoor tests for Fuji apple preservation (PDF)

## Notes

Data are available within the article or its supporting materials.
