# Supplementary material for: Combined Effects of Radiative and Evaporative Cooling on Fruit Preservation under Solar Radiation: Sunburn Resistance and Temperature Stabilization
Source: ACS Appl Mater Interfaces. 2022 Sep 29;14(40):45788–99. doi: 10.1021/acsami.2c11349 (PMC9562266; doi:10.1021/acsami.2c11349)
Supplement: Supplementary file 1 — am2c11349_si_001.pdf [file am2c11349_si_001.pdf]

Supporting Information

Combined Effects of Radiative and Evaporative Cooling for Fruit Preservation Under Solar Radiation: Sunburn Resistance and Temperature Stabilization

Liang Xu[a, b, c], Da-Wen Sun[a, b, c, d, *], You Tian[a, b, c], Libin Sun[a, b, c], Tianhao Fan[a, b, c], Zhiwei Zhu[a, b, c]

[a] School of Food Science and Engineering, South China University of Technology, Guangzhou 510641, China

[b] Academy of Contemporary Food Engineering, South China University of Technology, Guangzhou Higher Education Mega Center, Guangzhou 510006, China

[c] Engineering and Technological Research Centre of Guangdong Province on Intelligent Sensing and Process Control of Cold Chain Foods, & Guangdong Province Engineering Laboratory for Intelligent Cold Chain Logistics Equipment for Agricultural Products, Guangzhou Higher Education Mega Centre, Guangzhou 510006, China

[d] Food Refrigeration and Computerized Food Technology (FRCFT), Agriculture and Food Science Centre, University College Dublin, National University of Ireland, Belfield, Dublin 4, Ireland

[*] Corresponding author. E-mail: dawen.sun@ucd.ie; URLs: http://www.ucd.ie/refrig, http://www.ucd.ie/sun.

Method S1. Theoretical cooling power calculations

The net cooling power $P_{\text{net-rad}}$ of the NPs/NADES@PAAm/PVA is expressed as:

$$P_{\text{net-rad}}(T) = P_{\text{rad}}(T) - P_{\text{sol}} - P_{\text{atm}}(T_{\text{amb}}) - P_{\text{cond+conv}} \tag{7}$$

where the radiative power of NPs/NADES@PAAm/PVA is

$$P_{\text{rad}}(T) = \iint I_{\text{BB}}(\lambda)\varepsilon_{\text{aw}}(\lambda,\theta)cos\,\theta \text{d}\Omega\text{d}\lambda \tag{8}$$

where $\int \text{d}\Omega = 2\pi\int_0^{\pi/2}\text{d}\theta sin\theta$ is the angular integral over a hemisphere; $I_{\text{BB}}(\lambda)$ is the radiance of a blackbody at temperature T (T = 300 K), ε_{aw} is the emittance of NPs/NADES@PAAm/PVA in the atmospheric window. The absorbed power due to solar irradiation is

$$P_{\text{sol}} = \int I_{\text{sol}}(\lambda)A_{\text{sol}}(\lambda)\text{d}\lambda \tag{9}$$

where $A_{\text{sol}}(\lambda) = 1 - R_{\text{sol}}(\lambda)$ is the solar absorptance of NPs/NADES@PAAm/PVA. The absorbed power due to absorbed atmospheric radiation is

$$P_{\text{atm}} = \iint I_{\text{BB}}(\lambda, T_{\text{amb}})\varepsilon_{\text{atm}}(\lambda,\theta)\varepsilon_{\text{aw}}(\lambda,\theta)cos\,\theta \text{d}\Omega\text{d}\lambda \tag{10}$$

where $I_{\text{BB}}(\lambda, T_{\text{amb}})$ is the radiance of a blackbody at ambient temperature T_{amb} (T_{amb} = 300 K); ε_{atm} is the emittance of the atmosphere (air mass of 1.5 and water vapour column of 5.0 mm).[1] The dissipated power due to heat conduction and convection is

$$P_{\text{cond+conv}} = h_{\text{c}}(T_{\text{amb}} - T) \tag{11}$$

where h_{c} is the heat transfer coefficient.

Method S2. Water retention

$$\text{Water retention (\%)} = \frac{W_{\text{t}} - W_{\text{d}}}{W_{\text{t}}} \times 100 \tag{12}$$

where W_{t} and W_{d} are the weight of the swollen hydrogel at t time and the weight of the dried hydrogel, respectively.

Table S1. Detailed information for preparing different hydrogels

Study groups	NADES (*w/w*, NADES / mother liquor)	ZrO_2 NPs (*w/w*, ZrO_2 NPs / mother liquor)	PTFE NPs (*w/w*, PTFE NPs /mother liquor)
PAAm/PVA	0	0	0
NADES@PAAm/PVA	10%	0	0
NPs@PAAm/PVA	0	75%	75%
NPs/NADES@PAAm/PVA	10%	75%	75%

Table S2. Optimization of PTFE NPs and ZrO_2 NPs contents for NPs/NADES@PAAm/PVA with high solar reflectance.

Reflectance		ZrO_2 NPs content (%)				
		0	25	50	75	100
	0	0.02	0.77	0.78	0.80	0.82
PTFE NPs content (%)	25	0.78	0.81	0.85	0.85	0.87
	50	0.81	0.83	0.86	0.87	0.88
	75	0.82	0.85	0.87	0.89	0.89
	100	0.85	0.86	0.88	0.89	0.89

Table S3. Mechanical and swelling properties of the hydrogels with different treatments.

Study groups	Tensile stress (KPa)	Elongation at the break (%)	Swelling ratio (%)
PAAm/PVA	27.5	330.0	458.8
NADES@PAAm/PVA	15.0	446.1	473.2
NPs@PAAm/PVA	103.1	192.8	89.3
NPs/NADES@PAAm/PVA	82.5	256.2	98.8

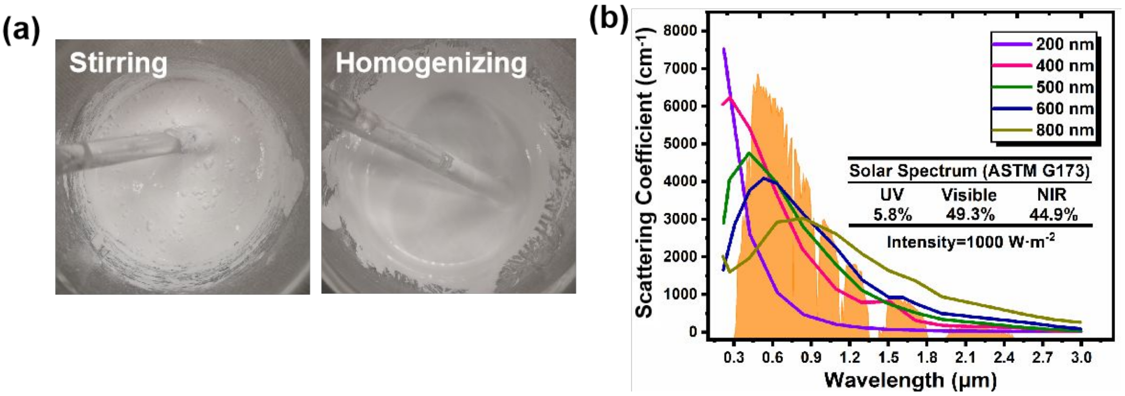

Figure S1. (a) Photos of hydrogel precursors with hand stirring or homogenizing treatment. (b) Scattering coefficient by ZrO_2 NPs with various sizes cited from Zhang's study.[2] The inset shows the contribution rate of different bands in the solar spectrum to the total solar irradiation.

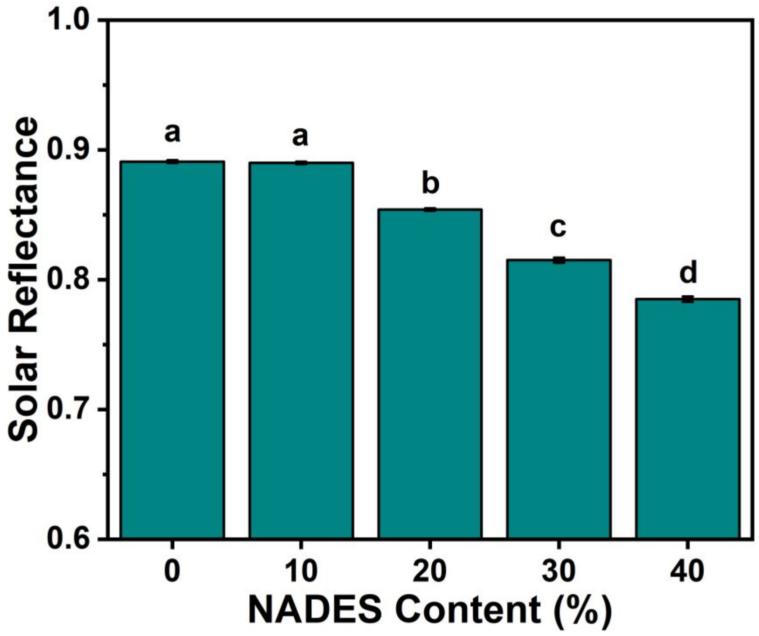

Figure S2. Optimization of different NADES concentrations for NPs/NADES@PAAm/PVA with high solar reflectance. Significance labels containing the same letter are not different by Duncan's test (p < 0.05).

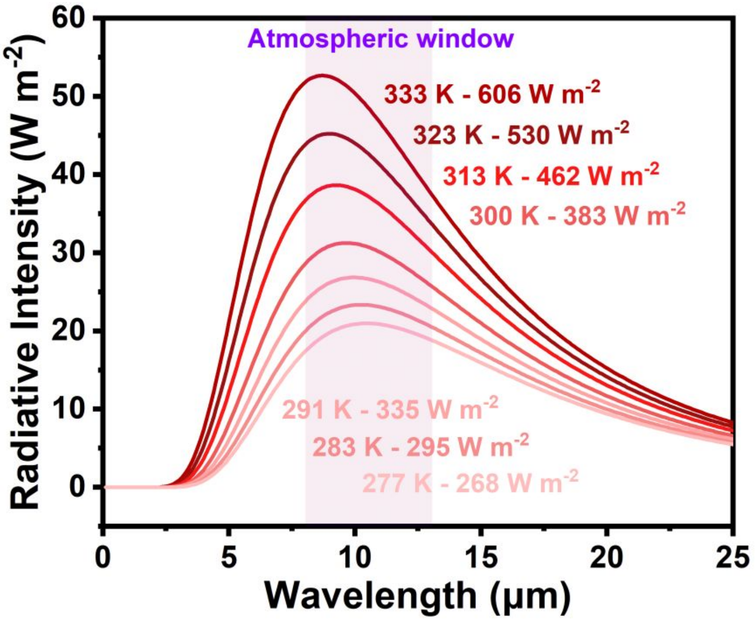

Figure S3. The radiative spectrum of a blackbody (ideal material) at different temperatures, and the corresponding total radiative powers.

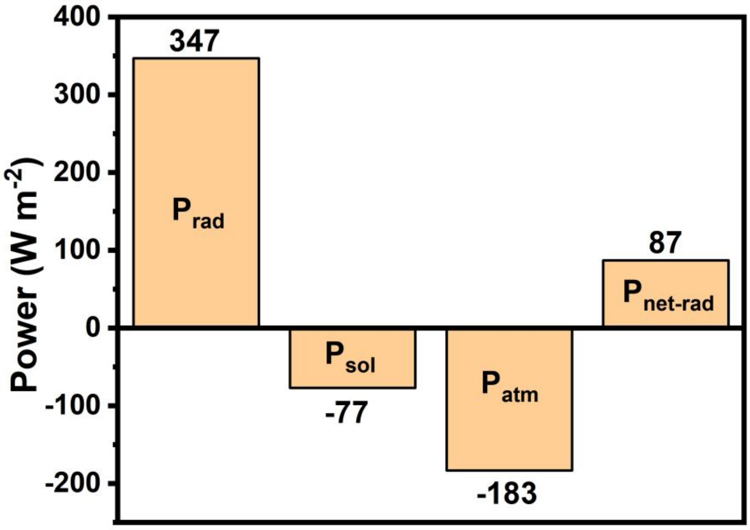

Figure S4. Power densities of thermal radiation (P_{rad}), absorbed solar (P_{sol}), absorbed atmospheric radiation (P_{atm}), and net radiative cooling ($P_{\mathrm{net\text{-}rad}}$) for NPs/NADES@PAAm/PVA according to the measured spectra.

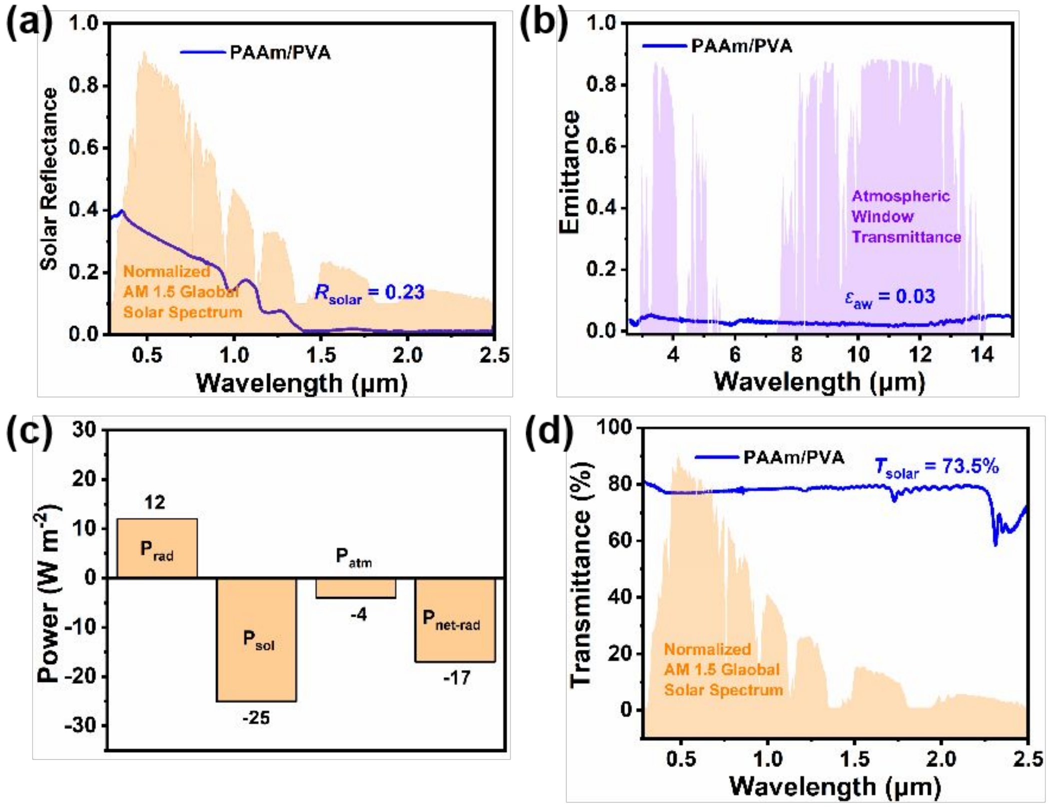

Figure S5. (a) Solar reflectance and (b) atmospheric emittance spectra of PAAm/PVA. (c) Power densities of P_{rad}, P_{sol}, P_{atm}, and $P_{\mathrm{net\text{-}rad}}$ for PAAm/PVA according to the measured spectra. (d) Solar transmittance of PAAm/PVA.

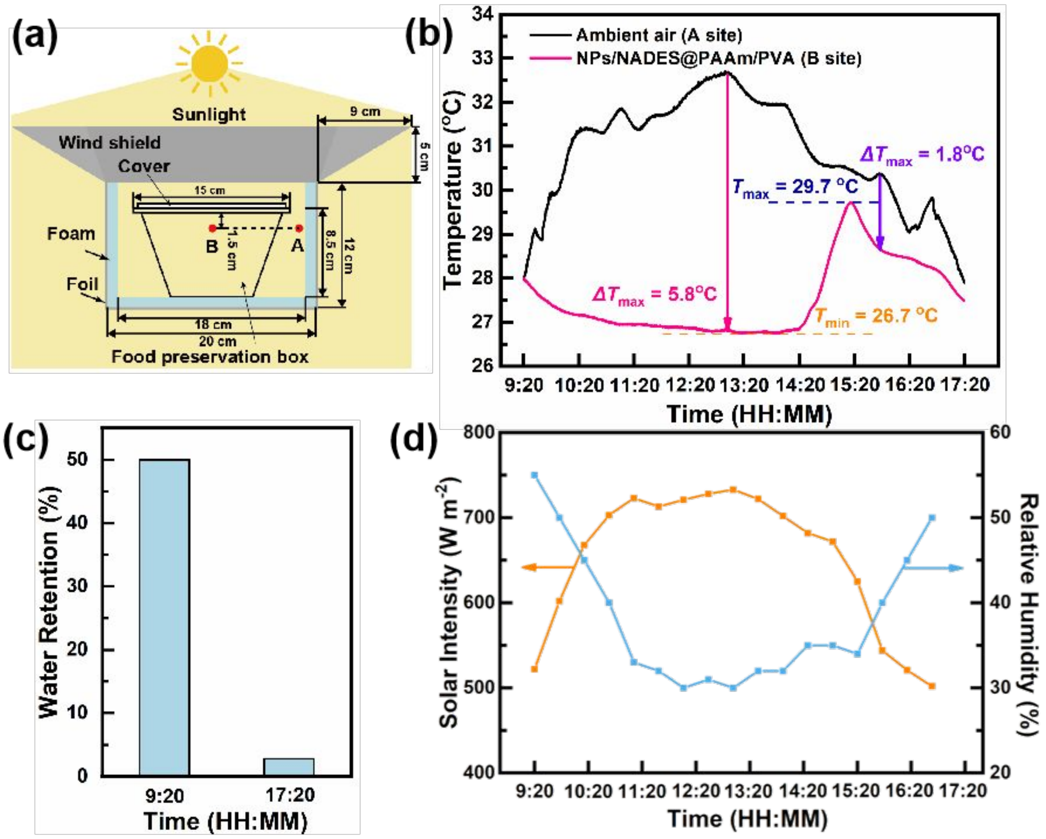

Figure S6. (a) Schematic of the custom-made device. The A and B sites represent the temperature of ambient air and food preservation box with NPs/NADES@PAAm/PVA, respectively. (b) Temperature of ambient air and the food preservation box with NPs/NADES@PAAm/PVA from 9:20 to 17:20 in Aug. 2022 in Guangzhou, China. (c) Changes in water retention of NPs/NADES@PAAm/PVA. (d) Real-time solar intensity and environment relative humidity.

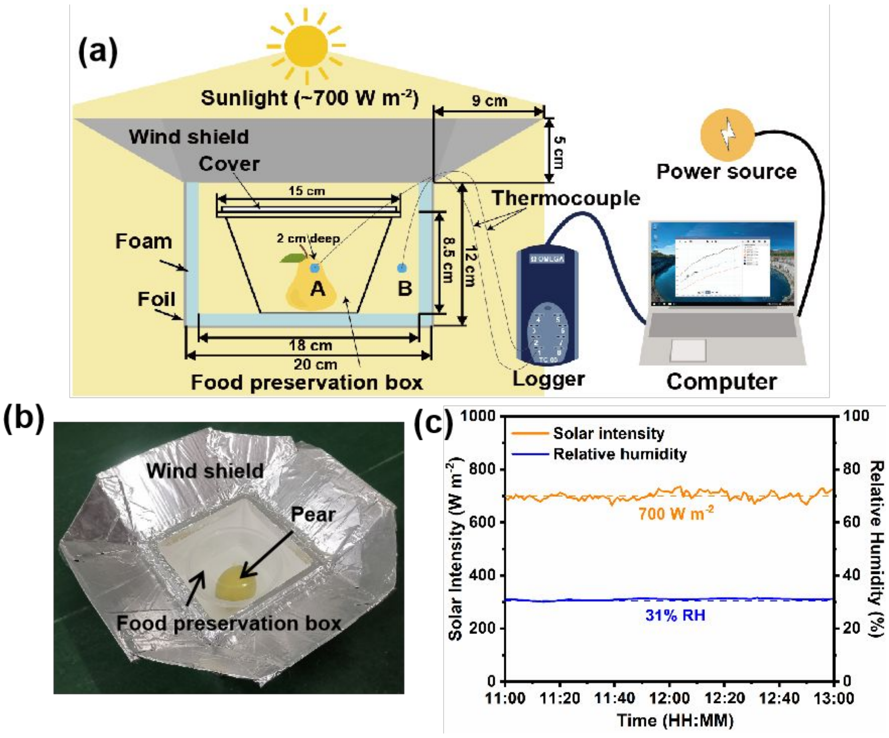

Figure S7. (a) Schematic of the custom-made device for real-time temperature determination. The A and B sites represent the temperature of fruit and ambient air, respectively. (b) Photograph of the custom-made device. (c) Real-time solar intensity and environment relative humidity for preservation experiment from 11:00 to 13:00 in Apr. 2022 in Guangzhou, China.

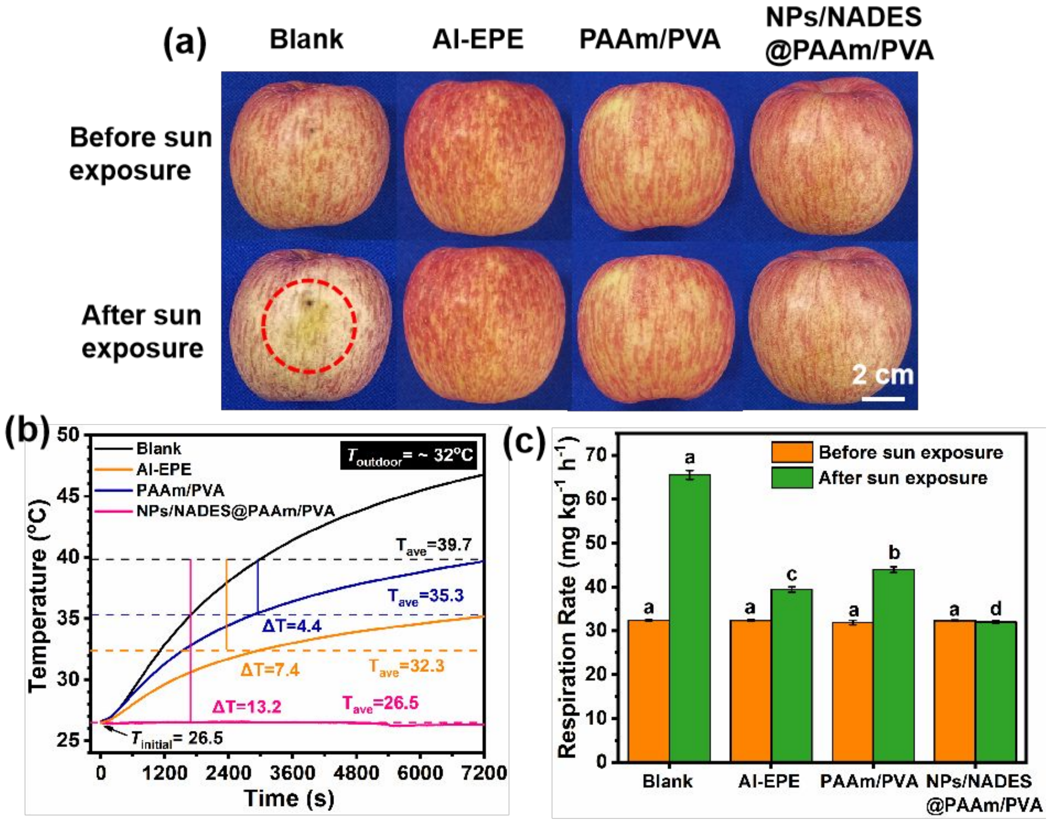

Figure S8. (a) Photographs showing Fuji apple with different treatments after sun exposure. (b) Temperature changes in Fuji apple with different treatments during sun exposure from 11:00 to 13:00 in Apr. 2022 in Guangzhou, China. (c) Respiration rates of Fuji apple with different treatments after sun exposure.

References

(1) IR Transmission Spectra, Gemini Observatory. http://www.gemini.edu/?q=node/10789 (accessed Nov 21, 2019).

(2) Zhang, Y.; Tan, X.; Qi, G.; Yang, X.; Hu, D.; Fyffe, P.; Chen, X., Effective Radiative Cooling with ZrO2/PDMS Reflective Coating. *Sol. Energy Mater. Sol. Cells* **2021,** *229*, 111129.
